# Nonunion of Fractures of the Ulna and Radius Diaphyses: Clinical and Radiological Results of Surgical Treatment

**DOI:** 10.5704/MOJ.1607.006

**Published:** 2016-07

**Authors:** H Boussakri, A Elibrahimi, M Bachiri, M Elidrissi, M Shimi, A Elmrini

**Affiliations:** * Department of Orthopeadic Surgery B4, Hassan II University Hospital, Fez, Morocco; ** Department of Orthopaedic and Traumatology Surgery, Midelt Hospital, Morocco

**Keywords:** Radius and ulna, diaphyseal fracture, nonunion

## Abstract

Aseptic nonunion of the radius and ulna is a major complication of forearm fractures, accounting for 2% to 10% of all forearm fractures. The aim of our study is to evaluate the functional and radiological results of surgical treatment of diaphyseal aseptic nonunion of the radius and ulna, with autologous bone grafting, decortication and internal plate fixation. A series of 21 patients (26 nonunions) was retrospectively reviewed, the average age was 35 years with a mean of 31,58 years (range 12-44 years). The fractures included isolated radius (n=6) and ulna (n=10), and both radius and ulna (n=5). The Grace and Eversmann score was used to evaluate our results. Fifteen had very good results, five good and one average. Consolidation of the two bones was attained in 6.2 months. Therefore, the functional prognosis of the upper limb imposes the need for an adequate treatment. This management strategy has enabled us to have satisfactory results. However, the best treatment of nonunion remains the preventive treatment with an optimal management and care of the forearm fractures.

## Introduction

Non unions are a major complication of diaphyseal fractures of the forearm, with eventual variable dysfunction of the upper limb and hand^1^. Non union is defined as absence of radiological and clinical signs of unions after an average period of six months. The use of dynamic compression plate has totally changed the prognosis of surgical treatment of diaphyseal fractures of the radius and ulna. Although large series in the literature have shown that this technique is simple with a low complication rate^1,2^, the incidence of aseptic nonunion of the forearm fractures remains significant between 2% and 10% in various publications^1,3-7^. The management of these non unions remains difficult due to the poor bone mass, the existence of previous implant material and joint stiffness that is associated with long-term immobilization^8^. The goal of surgery is to achieve complete union of the fractures and restore the functional anatomy between the radius and the ulna, so as to obtain a normal hand function^9^. This surgical stabilization at the nonunion should be associated with the compression of the fracture site and stimulation of bone formation by bone grafting and or decortication according to Judet *et al*^10^. Other treatment options are discussed, such as bone-marrow injection, and induced membrane technique which are not the choice of our surgeons.

In this single-centered retrospective study, we aim to analyze the causative factors of aseptic non union of the forearm fractures and evaluate the clinical and radiological results and the operative treatment with a dynamic compression plate, bone grafting and decortication.

## Materials and Methods

This is a retrospective study of 21 patients (with 26 nonunions) treated between May 2007 and January 2013 for aseptic diaphyseal nonunion of the radius and ulna. The inclusion criteria were the existence of aseptic nonunion of the diaphysis of the one and or both bones of the forearm treated with compression plate and screws, and associated with an autogenous iliac bone graft and osteomuscular decortication. Exclusion criteria were septic nonunions and metaphyseal-epiphyseal nonunions as well as nonunions restricted to the proximal or distal quarter fractures of both forearm bones and those treated with other therapeutic modalities.

We applied the classification of AO when we used the initial radiographs to classify fractures of the forearm^11^. Comparing the radiographs of delayed unions and nonunion of the forearm fractures, we noticed the absence of bone consolidation in the first stage after a period going from three to six months of the initial treatment; whereas, the radiographs of the second stage showed a total lack of union after a period of six months. On these radiographs, we also analyzed the level of nonunion, and its type as well as the initial treatment of the fracture of the forearm. Furthermore, the interpretation of this imagery also helped us search for technical errors and factors which would have contributed to nonunion.

Of these 21 patients (Table I), there were 16 men and five women, average age was 34.52 years, with extremes of 18 and 56, a standard deviation of 11.53 and a median of 34. Our center had initially taken care of six patients. We had 13 cases with fractures on the left side, and 8 cases on the right. Among our 21 patients, 12 patients had the fractures on their dominant side. Fifteen patients were engaged in manual work, four non-manual work and two had no vocations. All patients in our series had pain. On visual analogue scale, pain was an average of 7/10 (EV: 5-10)^12^. According to the topography of lesions: five cases had fractures of both bones of the forearm, 10 cases an isolated fracture of the ulna shaft, including two Monteggia fracture dislocations, and six cases with an isolated fracture of the radial shaft. We noted two cases of polytrauma (all following road traffic accidents) and three cases of open fracture (stage I in one case and stage II in two cases). One case of paresthesia in the territory of the median nerve had been recorded. However, there were no case of radial nerve palsy. The initial fracture line according to the AO classification of diaphyseal fractures of the forearm were five fractures A1 and two type A2, three A3, eight B1, six B3 and two C2 (Table I). The nonunion sites: 18 fractures in the middle third, six in the distal third, and two fractures in the proximal third. In eleven cases, the initial treatment of the fracture consisted of intramedullary pinning on by Kirschner wire, 14 plate and screws and an external fixation for the one open fracture.

**Table I:** Clinical and anatomical data of the 21 patients

Patient	Age/Sex	Nonunion localization	Type of fracture (AO)	Type of nonunion	Follow up (in months)
MM	18 M	Ulna	A1	oligotrophic	44
KA	56 M	Ulna	A1	oligotrophic	36
SA	34 F	Ulna	B3	oligotrophic	44
AM	43 M	Ulna	B3	oligotrophic	26
AT	24 M	Ulna	A1	hypertrophic	42
AE	28 F	Ulna	B3	hypertrophic	39
HA	31 F	Ulna	B1	hypertrophic	30
OF	37 M	Radius	B1	oligotrophic	32
OF	37 M	Ulna	B1	oligotrophic	32
EM	38 M	Radius	B1	oligotrophic	36
EJ	48 M	Radius	B3	oligotrophic	23
EJ	48 M	Ulna	B3	oligotrophic	23
HE	30 F	Ulna	B1	oligotrophic	34
HB	50 F	Radius	A2	hypertrophic	26
HB	50 F	Ulna	A2	hypertrophic	26
JE	38 M	Radius	A3	oligotrophic	44
BA	46 M	Radius	B1	oligotrophic	30
BA	46 M	Ulna	B1	oligotrophic	30
EK	30 M	Radius	B3	hypertrophic	17
JN	19 M	Radius	B3	hypertrophic	28
JD	27 M	Radius	C1	atrophic	12
JD	27 M	Ulna	C1	atrophic	12
BH	31 M	Ulna	A1	hypertrophic	46
AM	20 M	Ulna	A1	hypertrophic	37
DM	32 M	Radius	A3	oligotrophic	19
SM	27 M	Radius	B1	atrophic	24

AO : Muller classification of fractures. M : male, F : female.

The time between initial treatment and the treatment of nonunion was seven months (range: 5 to 16 months). Thus, three of our patients were operated before six months, which is theoretically considered as the period for diagnosis of nonunion. Conventionally, we differentiated between two types of nonunion: a viable nonunion (hypertrophic or oligotrophic) with a large callus or malunion that is mechanically incompetent, and atrophic nonunion (or devitalized nonunion) without callus, which required an osteogenic treatment. In our series, 58% of nonunions were oligotrophic (15 cases), 35% were hypertrophic (9 cases) and 7% were atrophic (2 cases) (Table I).

**Fig. 1:**
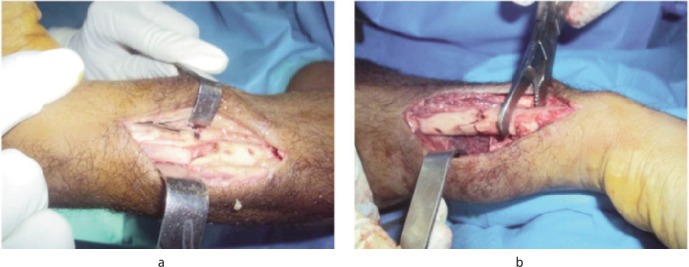
Intraoperative appearance. (a): nonunion of the ulna with the material fracture. (b) : decortication and debridement of the fracture site.

**Fig. 2:**
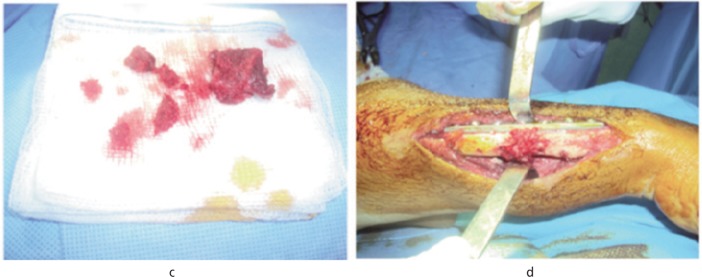
(c): corticocancellous graft. (d): final intraoperative appearance after implementation of graft and fixation by a dynamic compression (DCP).

**Surgical technique:** Based on the criteria of Corrales *et al.*^13^, the operating indications relied on the existence of clinical signs of nonunion (pain and / or mobility of the fracture) and radiological signs (lack of bone consolidation) after six months since the start of treatment of the initial fracture. The incision used was the classical anterior approach of Henry for the radius and, the dorsal approach centered on the ulnar ridge for the ulna. The first surgical step consisted of removing the osteosynthesis implant applied previously (Fig 1a), then the nonunion focal spot was clared of the fibrosis tissue and the tissue-ingrowth associated with medullary recanalization. Besides, we obtained the routine bacteriological samples and did an osteomuscular decortication (Fig 1b). The graft was then taken from the anterior ipsilateral iliac crest and packed opposite the nonunion focal spot (Fig 2a). Fixation with dynamic plate compression (type DCP (3.5mm)) was applied after manual compression of the nonunion focal spot. The optimum application included at least three screws on either side of the focal spot (Fig 2b). The upper limb was immobilized in a splint for 30 days and antibiotic prophylaxis instituted with first generation cephalosporin (1CG) for 48 hours. Functional rehabilitation (passive and active) of the proximal and distal joints was carried out. All bacteriological samples taken were negative.

The patients were followed-up to assess pain on analogue scale^12^, the range of motion of the elbow and wrist using a goniometer and detect any morbidity of bone graft site. We proceeded to an overall evaluation of our functional results through the Grace and Eversmann score^14^ and the DASH questionnaire^15^.

The post-operative radiological evaluation included AP and lateral views of the forearm. The consolidation was confirmed based on the existence of the two orthogonal evidence of bony bridges between the two ends of the nonunion focal spot, and absence of pain or tenderness at the fracture site. The radiological study was also to detect any evidence of malunion and to measure its angulation in the frontal and sagittal planes.

## Results

All patients were operated by two senior surgeons, specialized in upper limb surgery. For diaphyseal forearm aseptic nonunion with less than 3 cm of bone defect, were treated with debridement and fixation by a dynamic compression plate and iliac bone autograft. The average follow-up was 31.58 months (range: 12 to 44 months), the standard deviation was of 10.27, and the median was of 30.

The mean pre-operating range of elbow motion was 100° (70° - 140 °), with an average extension deficit of 10° and its average pronation 55° (0° -75°), with a supination of 50° (0° -85°). On the wrist, the average preoperative flexion was 48° (range: 10° - 90°), with an average extension of 60° (range: 15° - 90°). At the last following up, the average mobility of the wrist was 60° (20° - 90°) in flexion (postoperative improvement in comparison to the preoperative condition) and 70° (30° -90°) in extension; whereas, the average pronation was of 65° (0° - 80°) with supination of 70° (0° - 85°). The elbow average flexion was 130° (90° -140°) and the average extension deficit was 5°. Thus, we noted an improvement in postoperative mobility compared to the preoperative status (Table II -III).

**Table II:** Preoperative and postoperative range of motion of the series

	Preoperative period			Postoperative period		
Series (21 patients)	Mobility in pronation-supination	Mobility in Flexion-extension of the elbow	Mobility in Flexion-extension of the wrist	Mobility in pronation-supination	Mobility in Flexion-extension of the elbow	Mobility in Flexion-extension of the wrist
MM (Ulna)	75/80	130/0	70/90	80/80	135/0	65/80
KA(Ulna)	50/50	140/0	70/80	70/60	140/0	70/80
SA (Ulna)	40/55	135/0	70/80	40/60	130/0	70/80
AM (Ulna)	75/85	140/0	75/55	80/80	140/0	60/50
AT (Ulna)	70/55	120/0	70/80	80/50	120/-5	75/80
AE (Ulna)	75/70	110/0	50/55	80/80	140/0	75/80
HA (Ulna)	10/50	70/0	10/15	0/80	140/0	20/30
OF (Radius)	40/60	140/0	55/50	65/70	140/0	75/50
OF(Ulna)	40/60	140/0	55/50	65/70	140/0	75/50
EM(Radius)	80/80	140/0	75/90	75/50	130/0	70/80
EJ(Radius)	60/85	130/0	60 /60	70/70	140/0	70/60
EJ(Ulna)	60/85	130/0	60/60	70/70	140/0	70/60
HE (Ulna)	55/75	140/0	70/75	50/60	140/0	70/80
HB(Radius)	60/40	135/0	70/70	40/40	140/0	90/75
HB(Ulna)	60/40	135/0	70/70	40/40	140/0	90/75
JE(Radius)	70/70	140/0	60/90	75/80	120/0	70/80
BA(Radius)	0/30	90/0	55/55	55/80	140/0	80/80
BA(Ulna)	0/30	90/0	55/55	55/80	140/0	80/80
EK (Radius)	60/85	120/0	70/70	80/85	140/0	70/80
JN(Radius)	50/50	135/0	65/60	55/60	140/0	70/80
JD(Radius)	55/60	130/0	50/50	40/70	90/0	65/70
JD(Ulna)	55/60	130/0	50/50	40/70	90/0	65/70
BH(Ulna)	75/75	140/0	70/90	80/80	140/0	80/80
AM(Ulna)	60/70	130/0	80/80	70/50	120/0	75/80
DM (Radius)	40/20	140/0	50/60	50/0	140/0	55/75
SM (Radius)	75/70	135/0	70/90	80/80	130/0	70/75

**Table III:** The average preoperative and postoperative range of motion

**Average range of motion**	**Flexion (wrist)**	**Extension (wrist)**	**Pronation (forearm)**	**Supination (forearm)**	**Flexion (elbow)**	**Extension (elbow)**
Preoperative results	48°	60°	55°	50°	100°	Average
	[10° - 90°]	[15° - 90°]	[0° - 75°]	[0° - 85°]	[70° - 140°]	deficit of 10°
Postoperative results	60°	70°	65°	70°	130°	Average deficit
	[20° - 90°]	[30° - 90°]	[0° - 80°]	[0° - 85°]	[90°-140°)	of 5°

**Fig. 3:**
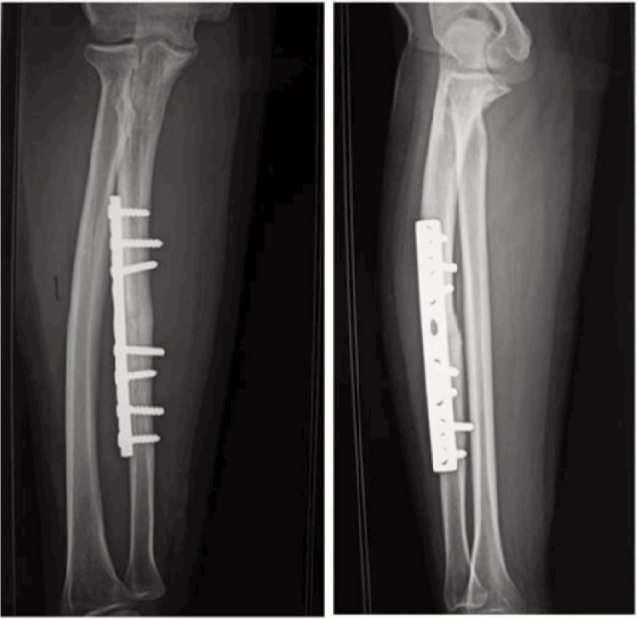
Nonunion of the ulna on a screwed plate after a period of 10 months with union of the focal spot.

**Fig. 4:**
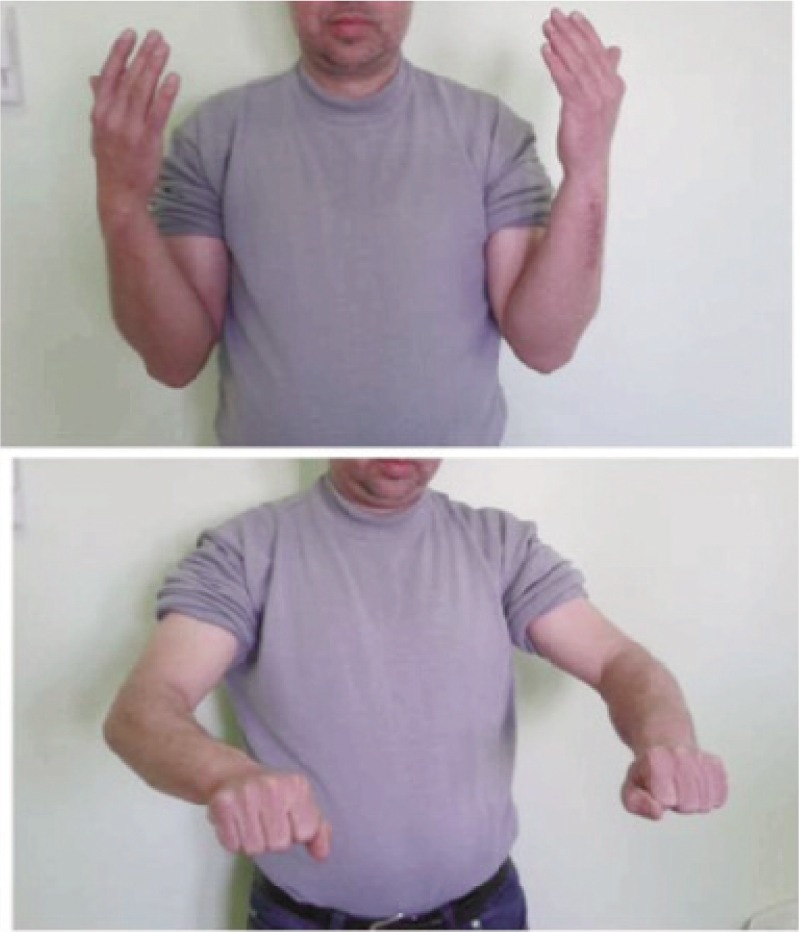
Functional results.

According to Grace and Eversmann score, the therapeutic results were: 15 excellent (Fig. 3-4), five good and one case of average results. This one case who presented with the average results, had been operated twice in another hospital. She was addicted to smoking, and she also had reflex sympathetic dystrophy syndrome. The overall average DASH score was 14 (5-36). Radiological consolidation was achieved in 25 nonunions (20 cases or 95.11%) within an average of 6.2 months. Nine cases had fracture consolidation in the first quarter, 13 cases in the second quarter and three cases in the third quarter, while one case had been consolidated in the fourth quarter. There was one case with radiological nonunion but was asymptomatic. The analysis of the AP and lateral radiographs (Fig. 3) allowed us to ascertain malunion without any functional disability in one case with callus on both bones of the forearm with varus of 2° on the radius and 3° on the ulna and two calluses with valgus of 2° on the radius. On the other hand, we did not find any limb length discrepancy or any rotational malunion.

### Complications:

We encountered two hematomas which had resorbed with local treatment and an early surgical site infection with methicillin resistant Staphylococcus aureus, which resolved with appropriate antibiotics therapy. A single case of chronic regional pain syndrome had been documented at one year follow-up.

The morbidity related the iliac crest was minimal with two patients who had mild pain, which did not require painkiller, and one unsightly scar.

## Discussion

Aseptic nonunion remains a significant late complication of diaphyseal forearm fractures with reported incidences ranging from 2% to 10%.^1, 3-7^.

Treatment of nonunion of the forearm remains a matter of debate. Several surgical techniques : internal fixation with bridging plate, intramedullary nailing, and external fixation have been recommended^17-22^. A successful surgical treatment of nonunion of the forearm requires several considerations: time to receive the appropriate care in relation to the initial injury, the number of previous surgery, the presence of infection, the length of the bone defect and finally the type of fixation method. The aim of the surgical treatment is to reestablish length of both the radius and ulna, restores their anatomy and quickly recovers the function of the upper limb and hand^21^. Diaphyseal fracture nonunion of upper limb, including the forearm, must be differentiated from diaphyseal nonunion of lower limb fractures because the main constraints are related to rotation and distraction and not to compression^23^. This fundamental constitutes the basis of diaphyseal fractures treatment of the forearm, which will block rigidly the shearing forces and rotation.

In the light of the results of our study and those reported in the literature (Table IV), the treatment of nonunion of diaphyseal forearm fractures by bone graft and fixation with a bridging plate gives excellent results if the principles of this technique are adhered. These principles include freshening the non-viable tissue, removal of the defective osteosynthesis material, restoration of alignment, length and rotation. We have found in our study that oligotrophic nonunion are more common than hypertrophic or atrophic nonunion, and that the high rate of nonunion for ulna is likely to be explained by the use of the intramedullary pinning to treat fractures of the ulna. Some authors have shown that stabilization of forearm fractures with intramedullary Kirschner wire and one-third tubular plate may have a high risk of nonunion because of the fastening failure^16^. On the other hand, no study has shown a significant difference in risk between ulna and radius that leads to nonunion^8-24^.

**Table IV:** Comparative results of the plate osteosynthesis in various series

**Authors**	**Number of cases**	**Radiological Union**	**Follow up (in months)**
Kloen P	47	100%	75
(2010)			[12-315]
Ring D	35	100%	43
(2004)			
Baldy dos Reis F(2009)	31	30/31 patients	43,2
		97 %	[24-72]
Our study	21	95%	32 (31,58)
			[12- 44]

Some authors report the importance of the use of intramedullary nailing in the treatment of nonunion of the forearm, a technique in which we have no experience, and we believe that this technique provides relative stability and lack of rotation control^21,31^. The locked intramedullary nail treatment is commonly used in the treatment of nonunions of long bones of the lower limb^32^. The authors emphasize on the possibility to cure nonunion of the forearm by an intramedullary nailing, profiting from closed focal spot fixation which would have union rates comparable to those using compression plates^21^. We think that we need to be more critical and do not advise the treatment of forearm nonunion by nailing, especially as some authors propose to associate an intramedullary nailing to a cortico cancellous bone graft with an open focal spot in order to improve anatomical results, particularly in case of atrophic nonunion^10,33^. In this case, we lose all the advantages of closed focal spot fixation; however, the locked nail seemed to be indicated only for hypertrophic diaphyseal nonunion without bone graft. Concerning the external fixation method, it is commonly used in the treatment of septic nonunion and its effectiveness is recognized. This type of treatment often use the Ilizarov external fixator^19^. Its proponents believe that through it they stop septic risks and periosteal devitalization, but in reality, it suffers from some side effects such as: difficulties in blocking rotation, obtaining an anatomical reduction, poor fixation and insufficient focal spot compression, as well as complications including nerve and vascular damage during the installation of sheets.

It is important to compare between the two types of nonunion, the one which only concerns one forearm bone and the one which concerns a dual radius and ulna nonunion whose impact on the function is definitely different.

The choice of bone graft is still a controversial subject^25,26^, since autologous bone graft is often performed in orthopaedic surgery for the treatment of nonunion, and even in the treatment of fractures of the forearm so as to accelerate healing as well as to prevent nonunion. This attitude remains controversial in the literature^4,5^. Furthermore, the iliac crest is the most common donor site for obtaining an autologous bone graft. These autografts have advantages, like the absence of risk of autoimmune response and disease transmission. Nicoll^17^ was the first to report the value of use of corticocancellous autograft in nonunion of the forearm, he recommended this technique in the absence of infection and the existence of a bone gap between the two fracture ends of less than 50mm^27^, this does not preclude that there are authors who believe that osteomuscular decortication is sufficient and can replace the bone graft since this latter can cause morbidity of the engraftment site^10^. Ramoutar *et al*^28^showed that the usual use of autologous bone graft was not necessary, and in their comparative study showed that the union ratio without the use of bone graft was 94.6% while adding bone graft let’s have a union ratio of 95%, without any difference (p = 0.67). This standard technique using a bone plate and an iliac graft is less effective in the treatment of long defects. It is particularly less effective in bone defects over 60 mm and which have operational difficulties for the management of the iliac graft so as to obtain sufficient compression and a normal length due to the physiological bowing (curvature) of the bone^27^. Ring *et al*^26^ showed that a non-vascularized autologous bone graft led to union in the case of atrophic nonunion with bone loss up to 6 cm while, Dos Reis *et al*^29^ showed, in a series of 31 patients, that treatment with corticocancellous bone graft and fixation with a plate for atrophic and hypertrophic nonunion led to excellent radiological and functional results. However, the treatment remains controversial for bone defects varying between 6cm and 10.5 cm^27,30^. Davey *et al* emphasized on the limits of the use indications concerning non-vascularized bone graft for bone defects exceeding 6 cm. In order to be successful, this surgical technique depends on the union and healing of corticocancellous bone graft.

Our results are in agreement with other reports published in the literature. We had only minimal complications and a satisfactory consolidation rate of 95% compared to the literature which varies between 91% and 100% (Table IV). We obtained excellent functional end results. Therefore, this surgical method is, from our point of view, an excellent technique to treat forearm diaphyseal fracture nonunion. Finally, the current therapeutic approach prevents the occurrence of nonunion. This rule is especially applied to fractures involving both bones of the forearm, which are conventionally treated by plate osteosynthesis. It seems clear that the absence of bone formation around the third or the fourth month pushes us to take an almost early preventive therapeutic approach with a possible bone intake and a change in the fixation if it seems essential.

## Conclusion

Surgical management steps for non union with decortication, bone autograft and stabilization with bridging plate has achieved satisfactory results in our series.

**Declaration of interest:** The authors declare that they have no conflicts of interest related to this article.
